# Kidney Function Tests and Continuous eGFR Decrease at Six Months after SARS-CoV-2 Infection in Patients Clinically Diagnosed with Post-COVID Syndrome

**DOI:** 10.3390/biomedicines12050950

**Published:** 2024-04-24

**Authors:** Madalina Boruga, Susa Septimiu-Radu, Prashant Sunil Nandarge, Ahmed Elagez, Gabriela Doros, Voichita Elena Lazureanu, Emil Robert Stoicescu, Elena Tanase, Roxana Iacob, Andreea Dumitrescu, Adrian Vasile Bota, Coralia Cotoraci, Melania Lavinia Bratu

**Affiliations:** 1 Department of Toxicology, Drug Industry, Management and Legislation, Faculty of Pharmacology, “Victor Babes” University of Medicine and Pharmacy Timisoara, 300041 Timisoara, Romania; madalina.boruga@umft.ro; 2 Doctoral School, “Victor Babes” University of Medicine and Pharmacy Timisoara, 300041 Timisoara, Romania; stoicescu.emil@umft.ro (E.R.S.); tanase.elena@umft.ro (E.T.); roxana.iacob@umft.ro (R.I.); bratu.lavinia@umft.ro (M.L.B.); 3 Department of General Medicine, D.Y. Patil Medical College Kolhapur, Kolhapur 416005, India; prashantnandarge1997@gmail.com; 4 Department of General Medicine, Misr University for Science & Technology, Giza 3236101, Egypt; ahmeddmahmouudd@gmail.com; 5 Third Discipline of Pediatrics, “Victor Babes” University of Medicine and Pharmacy Timisoara, 300041 Timisoara, Romania; gdoros@gmail.com; 6 Department XIII, Discipline of Infectious Diseases, “Victor Babes” University of Medicine and Pharmacy Timisoara, 300041 Timisoara, Romania; lazureanu.voichita@umft.ro; 7 Department of Radiology and Medical Imaging, “Victor Babes” University of Medicine and Pharmacy Timisoara, 300041 Timisoara, Romania; 8 Department of Anatomy and Embryology, “Victor Babes” University of Medicine and Pharmacy Timisoara, 300041 Timisoara, Romania; 9 Cardioprevent Foundation, 300134 Timisoara, Romania; andreea.dumitrescu@cardioprevent.org; 10 Multidisciplinary Doctoral School, “Vasile Goldis” Western University, 310025 Arad, Romania; bota.adrian1@yahoo.com (A.V.B.); rector_vg@uvvg.ro (C.C.); 11 Department of Hematology, Faculty of Medicine, “Vasile Goldis” Western University, 310025 Arad, Romania; 12 Center for Neuropsychology and Behavioral Medicine, Discipline of Psychology, Faculty of General Medicine, “Victor Babes” University of Medicine and Pharmacy Timisoara, 300041 Timisoara, Romania; 13 Center for Cognitive Research in Neuropsychiatric Pathology, Department of Neurosciences, “Victor Babes” University of Medicine and Pharmacy Timisoara, 300041 Timisoara, Romania

**Keywords:** COVID-19, SARS-CoV-2, long COVID, Infectious Disease

## Abstract

The long-term sequelae of SARS-CoV-2 infection are still under research, since extensive studies showed plenty of systemic effects of the viral infection, extending even after the acute phase of the infection. This study evaluated kidney function tests six months after SARS-CoV-2 infection in patients clinically diagnosed with Post-COVID Syndrome, hypothesizing persistent renal dysfunction evidenced by altered kidney function tests compared to baseline levels. Continuous eGFR decrease <30 at six months post-infection was considered the main study outcome. Conducted at the “Victor Babes” Hospital, this retrospective observational study involved adults with laboratory-confirmed SARS-CoV-2 infection and clinically-diagnosed Post-COVID Syndrome, excluding those with prior chronic kidney disease or significant renal impairment. Kidney function tests, including serum creatinine, blood urea nitrogen (BUN), estimated glomerular filtration rate (eGFR), alongside markers of kidney damage such as proteinuria and hematuria, were analyzed. Among 206 participants, significant differences were observed between the control (*n* = 114) and the Post-COVID group (*n* = 92). The Post-COVID group exhibited higher serum creatinine (109.7 μmol/L vs. 84.5 μmol/L, *p* < 0.001), lower eGFR (65.3mL/min/1.73 m^2^ vs. 91.2 mL/min/1.73 m^2^, *p* < 0.001), and elevated BUN levels (23.7 mg/dL vs. 15.2 mg/dL, *p* < 0.001) compared to the control group. Regression analysis highlighted significant predictors of continuous eGFR decrease <30 at six months post-infection. The development of acute kidney injury (AKI) during the initial COVID-19 illness emerged as a strong predictor of reduced eGFR (β = 3.47, *p* < 0.001). Additional factors, including a creatinine increase (23 μmol/L above the normal range) and an elevated Albumin to Creatinine Ratio (ACR) (>11 mg/g above the normal range), were significantly associated with eGFR reduction. Patients with Post-COVID Syndrome demonstrate significant renal impairment six months post-SARS-CoV-2 infection. The study’s findings stress the need for ongoing monitoring and intervention strategies for renal health in affected individuals, underscoring the persistent impact of COVID-19 on renal function.

## 1. Introduction

As the acute phase of the respiratory syndrome coronavirus 2 (SARS-CoV-2) infection has been extensively studied, attention has increasingly turned to the long-term sequelae of this virus, colloquially known as Post-COVID Syndrome or “Long COVID” [1,2]. This condition encompasses a wide range of persistent symptoms and clinical findings that continue for months beyond the initial infection, affecting multiple organ systems [3]. Among these, renal complications have emerged as a significant concern, given preliminary evidence suggesting that SARS-CoV-2 infection may precipitate or exacerbate kidney dysfunction [4].

Previous viral epidemics, such as those caused by the original SARS-CoV and the Middle East respiratory syndrome coronavirus (MERS-CoV), have been associated with acute kidney injury (AKI) [5,6,7]. Early data from the current pandemic have similarly highlighted kidney injury as a complication of acute COVID-19, especially in severe cases [8,9]. However, the persistence of renal dysfunction in the aftermath of SARS-CoV-2 infection, particularly in individuals with Post-COVID Syndrome, remains poorly characterized.

The concept of Post-COVID Syndrome encompasses a broad spectrum of symptoms and clinical outcomes, indicating a systemic impact that may involve direct viral injury, immune-mediated damage, or a combination of both [10,11]. While respiratory and cardiovascular sequelae have been the focus of much research, the potential for sustained kidney damage raises concerns about long-term renal health and the risk of chronic kidney disease (CKD) in these patients. Studies have reported that common COVID-19 symptoms such as common cold signs, cough, fever, sore throat, headache, anosmia, and dyspnea were more common in COVID-19 patients without renal disease [12,13,14]. However, understanding the trajectory of kidney function recovery or decline in the months following SARS-CoV-2 infection is critical for identifying individuals at risk and implementing timely interventions.

Kidney function tests, including serum creatinine, blood urea nitrogen (BUN), and estimated glomerular filtration rate (eGFR), are essential tools for assessing renal health. These biomarkers, alongside urinalysis for proteinuria and hematuria, offer insights into kidney damage and function [15,16,17,18]. Despite their importance, there is a paucity of data on the evolution of these parameters in the context of Post-COVID Syndrome, leaving a gap in knowledge that underscores the need for focused studies examining renal outcomes among survivors of SARS-CoV-2 infection.

Therefore, this study aims to evaluate kidney function tests measured at six months after SARS-CoV-2 infection in patients clinically diagnosed with Post-COVID Syndrome. We hypothesize that individuals with Post-COVID Syndrome exhibit persistent renal dysfunction, as evidenced by alterations in kidney function tests, compared to baseline levels. This research seeks to elucidate the extent of renal impairment in this patient population, contributing to a broader understanding of Post-COVID Syndrome’s long-term impacts and informing strategies for renal health monitoring and intervention in affected individuals.

## 2. Materials and Methods

### 2.1. Study Design and Ethics

This investigation was designed as a retrospective observational study aimed at evaluating kidney function tests in patients clinically diagnosed with Post-COVID Syndrome at approximately six months following SARS-CoV-2 infection. The database search and paper records search extended from January 2023 to December 2023, aligning with the phase where long-term effects of COVID-19 became increasingly recognized across the global medical community. Recruitment and data collection were centered around the “Victor Babes” Hospital affiliated with the “Victor Babes” University of Medicine and Pharmacy from Timisoara, Romania, integrating multidisciplinary expertise to address the complex needs of this patient population. The study received the approval number 2632 from 23 March 2022.

### 2.2. Patients’ Inclusion and Exclusion Criteria

Patients eligible for inclusion in this study were individuals aged 18 years or older who had a laboratory-confirmed SARS-CoV-2 infection, verified either by RT-PCR or rapid antigen tests. Additionally, these individuals must have been diagnosed with Post-COVID Syndrome, characterized by a constellation of symptoms persisting for more than 12 weeks after the acute infection, according to guidelines [19]. These symptoms include, but are not limited to, chronic fatigue, shortness of breath, cognitive disturbances (“brain fog”), joint pain, and ongoing loss of taste or smell, without any alternative diagnosis that could explain these symptoms [20].

Exclusion criteria were established to ensure the safety of participants and the integrity of the study data. Patients were excluded if they had a history of chronic kidney disease or significant renal impairment prior to SARS-CoV-2 infection, as indicated by medical records or baseline kidney function tests showing an eGFR below 60 mL/min/1.73 m^2^. Additional exclusion factors included acute renal failure or dialysis within the six months preceding the study, significant cardiovascular disease (e.g., recent myocardial infarction, unstable angina, uncontrolled arrhythmias), severe psychiatric conditions impacting consent or participation ability, and current pregnancy or breastfeeding.

Patients receiving immunosuppressive therapy at the time of SARS-CoV-2 infection or those with a known history of autoimmune diseases affecting kidney function were also excluded. This was to minimize confounding variables that could influence renal function independently of Post-COVID Syndrome. Consent was a prerequisite; thus, individuals who did not agree to participate in the study or who were unable to provide informed consent due to cognitive impairments were excluded.

The study aimed to create a well-defined cohort that accurately represents the spectrum of Post-COVID Syndrome while ensuring that the focus remained on the effects of the condition on kidney function, free from interference by pre-existing renal conditions or other comorbidities that could confound the results. Thus, a control group of patients with COVID-19 who did not develop Post-COVID Syndrome was also included as comparison with the study group, in order to serve as a reference for baseline characteristics of COVID-19 patients.

### 2.3. Study Variables

The study variables were categorized as follows: (1) Demographic and clinical variables: age, gender, and body mass index (BMI). Additionally, data on the severity of initial SARS-CoV-2 infection, comorbidities, and vaccination status were collected to evaluate their potential impact on Post-COVID Syndrome and kidney function. (2) Laboratory data: key to this study were renal function tests, including serum creatinine, blood urea nitrogen (BUN), and estimated glomerular filtration rate (eGFR), alongside markers of kidney damage such as proteinuria and hematuria assessed through urinalysis. Complementary to these were general health indicators like complete blood count (CBC), electrolytes, C-reactive protein (CRP) for inflammation, and other relevant markers such as ferritin and interleukin-6 (IL-6) to evaluate systemic involvement and inflammation. (4) Symptomatology and Post-COVID Syndrome assessment: a structured assessment of Post-COVID Syndrome symptoms, focusing on duration, severity, and the impact on daily living, was performed. This included documentation of specific symptoms relevant to renal function, such as edema and changes in urination patterns, alongside a comprehensive review of systemic symptoms such as fatigue, cognitive dysfunction, and myalgia. (5) Healthcare utilization and intervention data: information on any interventions, medications related to kidney function or Post-COVID symptoms, and any renal-specific consultations or treatments received during the study period were recorded. This also included data on follow-up visits, hospital readmissions, and any outpatient care specific to renal health or Post-COVID management.

The current study utilized the Chronic Kidney Disease Epidemiology Collaboration (CKD-EPI) Creatinine Equation (2021) for eGFR calculation [21]. This method exclusively uses serum creatinine as the biomarker, adjusted for age, sex, and race, and is widely recognized for its accuracy across varying levels of kidney function.

In our study, COVID-19 severity was characterized according to guidelines [22], based on clinical and laboratory criteria as follows: (1) Mild COVID-19, with the presence of symptoms like fever, cough, sore throat, malaise, headache, muscle pain without shortness of breath, dyspnea, or abnormal chest imaging; (2) Moderate COVID-19 with the evidence of lower respiratory disease during clinical assessment or imaging and a saturation of oxygen (SpO_2_) ≥94% on room air at sea level; (3) Severe COVID-19, showing a respiratory frequency >30 breaths per minute, blood oxygen saturation ≤93% on room air at sea level, ratio of arterial partial pressure of oxygen to fraction of inspired oxygen (PaO_2_/FiO_2_) <300, or lung infiltrates >50% within 24 to 48 h.

### 2.4. Statistical Analysis

The statistical processing of data in this study was conducted using the SPSS software, version 26.0. The patient groups were matched by age, gender, and COVID-19 severity. To describe the demographics and clinical characteristics of the participants, descriptive statistics were utilized. This involved summarizing continuous variables by their mean values and standard deviations and representing categorical variables through their counts and percentages. For evaluating differences in categorical data, the Chi-square test and Fisher’s exact test were applied as appropriate. Analysis of differences in continuous variables that did not adhere to a normal distribution was carried out using the Mann-Whitney U test. In cases where continuous variables were normally distributed, comparisons were made using the Student’s *t*-test for independent samples, reporting mean values and standard deviations. For non-normally distributed data, comparisons of median values and interquartile ranges (IQR) were made with the Mann-Whitney U test. All statistical tests were two-tailed, with a significance level set at a *p*-value of less than 0.05, indicating statistical significance.

## 3. Results

### 3.1. Patients’ Background

In the current study a detailed demographic analysis was conducted to assess various health and lifestyle variables between the control group (*n* = 114) and the post-COVID group (*n* = 92). The average age of participants in the control group was 55.2 years (±8.5), while the post-COVID group had an average age of 56.9 years (±7.6), with the difference not being statistically significant (*p* = 0.136). Gender distribution was also compared, showing a slight difference in proportions with men constituting 51.8% of the control group and 54.3% of the post-COVID group, but this difference was not statistically significant (*p* = 0.710).

There were no significant changes in Body Mass Index (BMI) between the two study groups (*p* = 0.900). The analysis of COVID-19 vaccination status revealed a higher percentage of individuals in the post-COVID group (41.3%) receiving ≥2 doses compared to the control group (32.5%), though the difference was not statistically significant (*p* = 0.317). Antiviral medication requirement showed a higher percentage in the post COVID group (71.7%) compared to the control group (59.6%); [not significant (*p* = 0.096)].

No significant differences were observed in the severity of COVID-19 between the groups (*p* = 0.909) or in personal history variables such as smoking and Charlson Comorbidity Index (CCI) greater than 2 (*p* = 0.851 and *p* = 0.904, respectively). However, notable differences were observed in kidney injury outcomes. The post-COVID group had a significantly higher incidence of acute kidney injury (AKI) during admission (17.4%) compared to the control group (6.1%, *p* = 0.014). Nevertheless, none of the 23 patients who developed AKI required renal replacement therapy during admission. Furthermore, a significant proportion of the post-COVID group experienced a decrease in eGFR below 30 from baseline (27.2%) compared to the control group (9.6%, *p* = 0.001). Additionally, continuous eGFR decrease below 30 at six months was observed exclusively in the post-COVID group (23.9%), as presented in Table 1.

### 3.2. Laboratory Data

The white blood cell count, a marker of infection and inflammation, was significantly higher in the Post-COVID group (16.6 ± 3.4 1000/mm^3^) compared to the control group (11.8 ± 1.7 1000/mm^3^), with a *p*-value of <0.001, indicating a substantial increase beyond the normal range (4.5–11.0 1000/mm^3^). Similarly, levels of C-reactive protein (CRP), an acute-phase reactant indicative of inflammation, were markedly elevated in the Post-COVID group (78.2 ± 24.6 mg/dL) compared to the control group (23.1 ± 2.8 mg/dL), with the difference being statistically significant (*p* < 0.001). Lymphocyte counts, which are critical for the immune response, were significantly lower in the Post-COVID group (1.3 ± 0.6 1000/mm^3^) than in the control group (2.7 ± 0.8 1000/mm^3^), falling below the normal range (1.0–4.8 1000/mm^3^), with a *p*-value of <0.001.

Significant elevations were also observed in liver enzymes, including aspartate aminotransferase (AST) and alanine aminotransferase (ALT), in the Post-COVID group (47.3 ± 11.8 U/L and 53.7 ± 14.3 U/L, respectively) compared to the control group (21.5 ± 5.2 U/L and 18.6 ± 7.2 U/L, respectively), both with *p*-values of <0.001. These increases surpass the normal ranges (10–40 U/L for AST and 7–35 U/L for ALT).

Further analysis revealed significant increases in IL-6 (44.6 ± 19.7 pg/mL), procalcitonin (0.48 ± 0.22 ug/L), D-dimers (498.5 ± 198.4 ng/mL), and ferritin (607.5 ± 295.7 ng/mL) levels in the Post-COVID group compared to the control group, all with *p*-values of <0.001. Hemoglobin levels, vital for oxygen transport, were lower in the Post-COVID group (13.4 ± 1.4 g/dL) compared to the control group (14.7 ± 1.3 g/dL), with the difference being statistically significant (*p* < 0.001) and indicating potential anemia in the Post-COVID group within the context of normal ranges (13.0–17.0 g/dL), as presented in Table 2.

Significant improvements were noted across several blood parameters when comparing values at admission with those six months post-admission. The white blood cell count, initially elevated (16.6 ± 3.4 1000/mm^3^) beyond the normal range, decreased significantly to within normal limits (10.9 ± 2.3 1000/mm^3^, *p* < 0.001). Similarly, lymphocyte counts showed a notable improvement from a reduced count (1.3 ± 0.6 1000/mm^3^) at admission to a healthier level (2.4 ± 0.7 1000/mm^3^, *p* < 0.001) within the normal range (1.0–4.8 1000/mm^3^), suggesting recovery of immune function. Liver function tests, including aspartate aminotransferase (AST) and alanine aminotransferase (ALT), initially showed marked elevation (47.3 ± 11.8 U/L and 53.7 ± 14.3 U/L, respectively) indicating liver stress or injury. These levels significantly improved to 29.4 ± 7.5 U/L and 24.8 ± 9.1 U/L, respectively, both within the normal ranges (10–40 U/L for AST and 7–35 U/L for ALT), with *p*-values of <0.001.

Markers of inflammation and infection, such as C-reactive protein, interleukin-6, and procalcitonin, also demonstrated significant reductions. CRP levels decreased from 78.2 ± 24.6 mg/dL to 14.7 ± 9.3 mg/dL, IL-6 from 44.6 ± 19.7 pg/mL to 12.7 ± 4.6 pg/mL, indicating a substantial reduction in systemic inflammation. Additionally, D-dimers and ferritin levels, which were elevated at admission (498.5 ± 198.4 ng/mL and 607.5 ± 295.7 ng/mL, respectively) improved significantly to 295.3 ± 102.7 ng/mL and 248.3 ± 149.2 ng/mL, respectively, with *p*-values of <0.001, although D-dimers remained above the normal range (< 250 ng/mL), as described in Table 3.

Creatinine levels were significantly higher in the Post-COVID group (109.7 ± 16.4 μmol/L) compared to the control group (84.5 ± 11.7 μmol/L), with a *p*-value of <0.001, suggesting reduced kidney function in the former. This was further corroborated by the eGFR, which was substantially lower in the Post-COVID group (65.3 ± 12.8 mL/min/1.73 m^2^) than in the control group (91.2 ± 7.3 mL/min/1.73 m^2^), with a *p*-value of <0.001, indicating significant kidney impairment. BUN levels, another key marker of kidney function, were also significantly higher in the Post-COVID group (23.7 ± 7.2 mg/dL) compared to the control group (15.2 ± 3.6 mg/dL), with a *p*-value of <0.001.

Proteinuria was significantly more pronounced in the Post-COVID group (198.3 ± 91.2 mg/dL) versus the control group (105.1 ± 48.6 mg/dL), with a *p*-value of <0.001. Similarly, albuminuria and the ACR, were markedly higher in the Post-COVID group (52.4 ± 18.9 mg/g and 59.1 ± 23.6 mg/g, respectively) than in the control group (22.7 ± 8.2 mg/g and 24.8 ± 10.1 mg/g, respectively), both with *p*-values of <0.001. Hematuria was significantly more common in the Post-COVID group (3.8 ± 2.1 cells/HPF) compared to the control group (1.5 ± 1.2 cells/HPF), with a *p*-value of <0.001. Urine specific gravity, which measures the concentration of substances in the urine, was lower in the Post-COVID group (1.016 ± 0.008) compared to the control group (1.021 ± 0.004), with a *p*-value of <0.001. Sodium and potassium levels showed no significant differences (Table 4).

Significant improvements were observed in several kidney function parameters over the six-month period. Creatinine levels, which were initially elevated (109.7 ± 16.4 μmol/L) well above the normal range (60–110 μmol/L), indicating impaired kidney function, decreased significantly to 90.4 μmol/L (*p* < 0.001), approaching the upper limit of the normal range and suggesting partial recovery of renal function. The eGFR improved from 65.3 mL/min/1.73 m^2^ to 70.6 mL/min/1.73 m^2^ (*p* = 0.002). Although this improvement signifies enhanced kidney function, the eGFR values remained below the normal threshold (>90 mL/min/1.73 m^2^), indicating and ongoing reduced renal impairment. BUN levels decreased significantly from 23.7 mg/dL to 18.3 mg/dL (*p* < 0.001).

Markers of renal damage, such as proteinuria, albuminuria, and the ACR, all showed significant decreases, indicating a reduction in kidney damage over time. Improvements were also noted in urine specific gravity and osmolality, both of which returned to within or closer to their normal ranges, indicating better kidney concentrating ability. Furthermore, sodium and potassium levels showed significant normalization, with sodium increasing from 138.2 mmol/L to 140.1 mmol/L (*p* < 0.001) and potassium decreasing from 4.7 mmol/L to 4.5 mmol/L (*p* = 0.007), as presented in Table 5.

The findings revealed significant differences between the two groups, indicating ongoing renal impairment in the Post-COVID group six months after discharge. Creatinine levels were significantly higher in the Post-COVID group (90.4 ± 13.6 μmol/L) compared to the control group (84.5 ± 11.7 μmol/L) with a *p*-value of 0.001, still within the normal range (60–110 μmol/L) but indicative of reduced kidney function. The estimated eGFR was significantly lower in the Post-COVID group (70.6 ± 11.1 mL/min/1.73 m^2^) than in the control group (91.2 ± 7.3 mL/min/1.73 m^2^), with a *p*-value of <0.001.

BUN levels were also significantly higher in the Post-COVID group (18.3 ± 5.8 mg/dL) compared to the control group (15.2 ± 3.6 mg/dL), with a *p*-value of <0.001, indicating a degree of renal stress or impairment. Proteinuria, the presence of excess proteins in the urine, was markedly higher in the Post-COVID group (155.9 ± 65.4 mg/dL) than in the control group (105.1 ± 48.6 mg/dL), with a *p*-value of <0.001.

Hematuria and proteinuria were significantly more prevalent in the Post-COVID group (2.0 ± 1.7 cells/HPF and 38.8 ± 15.2 mg/g, respectively) compared to the control group (1.5 ± 1.2 cells/HPF and 22.7 ± 8.2 mg/g, respectively), with *p*-values of 0.014 and <0.001, respectively. The ACR was also significantly elevated in the Post-COVID group (39.5 ± 16.3 mg/g) compared to the control group (24.8 ± 10.1 mg/g), with a *p*-value of <0.001 (Table 6).

### 3.3. Risk Assessment

Severe COVID-19 severity showed a significant positive correlation with eGFR decrease, having a β of 2.05 (*p* = 0.001), suggesting that patients who had severe COVID-19 are more likely to experience a notable decline in kidney function, with the CI stretching from 1.89 to 4.21.

**Figure 1:**
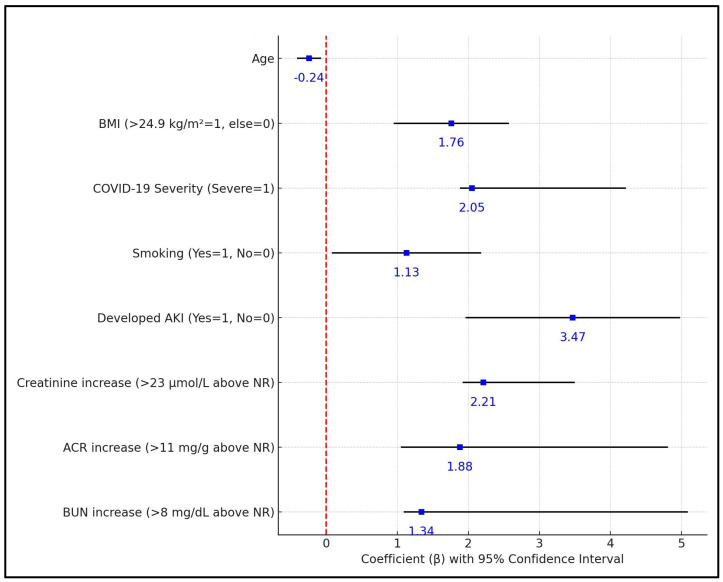
Regression analysis results of factors predicting continuous eGFR decrease <30 at six months post SARS-CoV-2 infection.

AKI was a strong predictor of eGFR decline, with a β of 3.47 (*p* < 0.001) and a CI of 1.97 to 4.97, clearly demonstrating that patients who developed AKI during their initial COVID-19 illness had a significantly higher risk of sustained reduction in kidney function. An increase in creatinine levels of more than 23 μmol/L above the normal range was also a significant predictor of eGFR decline, with a β of 2.21 (*p* < 0.001) and a CI of 1.93 to 3.49, highlighting the impact of kidney injury marked by elevated creatinine on long-term kidney function. Increases in the ACR greater than 11 mg/g above the normal range and BUN increases greater than 8 mg/dL above the normal range were also significantly associated with decreased eGFR, with β coefficients of 1.88 (*p* < 0.001, CI: 1.06 to 4.80) and 1.34 (*p* = 0.001, CI: 1.10 to 5.08), respectively (Table 7, Figure 1).

## 4. Discussion

### 4.1. Literature Findings

The study’s findings pointed to significant kidney impairment in Post-COVID patients, especially when noting elevated creatinine levels and reduced eGFR compared to controls. This distinct pattern suggests COVID-19’s unique impact on renal health, beyond what’s typically observed in acute illness. While some recovery was noted over six months, evidenced by improvements in eGFR and reductions in proteinuria and albuminuria, the continuous abnormalities highlight the risk of developing chronic kidney disease post-COVID. Our analysis revealed significant differences in inflammatory biomarkers between the POST COVID group and the Control group, as detailed in Table 2. This observation aligns with the findings presented in Table 7, where the severity of COVID-19 infection was correlated with a sustained decrease in eGFR to levels below 30 mL/min/1.73 m^2^ at six months post-infection. These results suggest that the inflammatory response elicited by severe COVID-19 may have a prolonged impact on renal function. Despite the partial recovery observed in some kidney function metrics, the persistence of certain abnormalities underlines the potential for long-lasting renal impairment. This raises concerns about the capacity of the kidneys to fully recover after severe COVID-19, pointing to a need for ongoing research into therapeutic strategies that could support renal healing and prevent chronic conditions.

The study findings mirrored those reported by Tannor et al. [23], emphasizing the exacerbated risks and outcomes of COVID-19 on individuals with pre-existing kidney conditions, particularly underlining the severe consequences in low-income settings due to inadequate healthcare infrastructure. Both studies underscored the heightened incidence of acute kidney injury and the progression to chronic kidney disease post-COVID-19 infection, pointing out the global inequities in managing these complications. Similarly, Brogan et al. [24] identified acute kidney injury and potential exacerbation of chronic kidney diseases as critical concerns, noting the unclear role of direct viral infection of kidney cells in these processes. They also highlighted the increased mortality associated with kidney conditions in the context of COVID-19, especially among those with severe disease or on replacement therapy, underscoring the gap in clinical trials for COVID-19 therapies that include patients with severe kidney disease.

Similarly, Žulpaitė et al. [25] reported a significant association between COVID-19 and renal injury, noting that patients with acute kidney injury or chronic kidney disease exacerbated by AKI had higher mortality rates, up to 7.81 times higher for those with AKI and CKD combined compared to those with CKD alone. This study’s findings of elevated creatinine levels and reduced eGFR six months post-COVID further underscore the lingering renal impairment, closely echoing Žulpaitė et al.’s [25] observation that COVID-19 patients with renal complications faced longer hospital stays and increased likelihood of death. Mahalingasivam et al. [26] highlighted the global challenge of researching kidney diseases in the COVID-19 context, pointing to variabilities in SARS-CoV-2 testing access and the incidence of infection, complicating the clear classification and management of AKI and CKD. The disparities and methodological hurdles emphasized by Mahalingasivam et al. [26] underline the necessity for a nuanced understanding of COVID-19’s impact on renal health, suggesting a convergence in findings across studies that COVID-19 significantly exacerbates risks for patients with kidney diseases, further complicating their prognosis and care.

The study findings, when considered alongside other research provide a comprehensive understanding of COVID-19’s impact on renal health, even though the majority of existing studies did not report on the association of CKD with Post-COVID syndrome. Jdiaa et al. [27] highlighted the significantly higher risks of hospitalization and mortality among COVID-19 patients with chronic kidney disease, indicating a hospitalization risk ratio of 1.63 and a mortality hazard ratio of 1.48 for these patients. This correlates with the observed persistent renal impairment in post-COVID patients in the current study, emphasizing the virus’s severe effects on individuals with pre-existing renal conditions. Martin de Francisco et al. [28] delved into the categorization of COVID-19 and its long-term manifestations, including persistent symptoms known as long COVID, which has been associated with severe disease phases and pre-existing conditions such as CKD. They also noted a lack of comprehensive renal data in post-COVID syndrome discussions, suggesting a gap in recognizing and addressing renal impairments in these patients.

The findings of the current study, in concert with those presented by Copur et al. [29] and La Porta et al. [30], shed light on the intricate relationship between COVID-19 and renal function. Copur et al., emphasize the significant renal involvement during and post-COVID-19 infection, highlighting a notable decline in renal function up to 12 months following the infection, even in patients who did not exhibit acute kidney injury initially. This observation aligns with the current study’s findings, where persistent renal impairment was evident six months post-COVID, suggesting a prolonged impact of the virus on kidney health. La Porta et al., through a retrospective study, identified AKI as a critical factor associated with increased mortality and severity of COVID-19, corroborating the significant role of renal dysfunction in the disease’s outcomes as observed in the current study. However, La Porta et al., also noted that baseline kidney function mitigated the role of age in COVID-19 severity and mortality, offering a nuanced view of the interplay between renal function and other risk factors.

Akliu et al.’s research [31] demonstrated a notable decrease in major adverse kidney events (MAKE) among patients with COVID-19-associated AKI, with an adjusted hazard ratio for MAKE of 0.67, indicating a significantly lower risk when compared to AKI from other illnesses. This was further supported by a marked reduction in all-cause mortality, with an adjusted hazard ratio (aHR) of 0.31, and a lower rate of worsened kidney function (aHR, 0.78) in the COVID-AKI group. This suggests that despite the initial severity, patients with COVID-19-associated AKI may have a somewhat more favorable long-term kidney prognosis than those with AKI due to other causes. In contrast, Lin et al. [32] investigated the genetic links between CKD and critical COVID-19, finding a significant association with an odds ratio of 1.28 for the risk of severe COVID-19 in individuals with genetically predicted CKD. However, they did not find a meaningful correlation between severe COVID-19 and CKD when using genome-wide significant SNPs as instrumental variables for critical COVID-19 (OR = 1.03), indicating the complexity of the relationship between COVID-19 severity and underlying CKD. Together, these studies highlight the nuanced interplay between COVID-19 and renal health, suggesting both a direct impact of the virus on kidney function and a genetic predisposition that may influence disease severity.

To further enhance the understanding of the renal impacts of COVID-19, particularly in severe cases, it is crucial to consider the differential clearance of creatinine and cystatin C. Creatinine, with a molecular weight of 0.11 kDa, and cystatin C, at 13 kDa, provide insights into changes in glomerular filtration barrier properties, potentially indicative of “shrunken pore syndrome” (SPS). SPS, characterized by selective dysfunction in glomerular filtration, may be a critical factor in the renal pathology observed with severe COVID-19. The study by Herget-Rosenthal et al. [33] explored how alterations in the filtration of molecules of different sizes can signal changes in kidney barrier function, relevant in the context of systemic diseases such as COVID-19. However, a 2022 study revealed that in ICU-treated patients, dexamethasone influenced the estimated glomerular filtration rate measured by cystatin C but not by creatinine, suggesting that steroid treatment could impact cystatin C levels and thereby eGFR estimates [34].

Therefore, the current study stands along the existing literature on the COVID-19 long-term outcomes on kidney function, underscoring the necessity of incorporating renal health into the long-term care plan for COVID-19 survivors. With specific risk factors identified, healthcare providers can better target those most at risk of sustained kidney damage, offering a more strategic approach to post-COVID care. The study not only adds to our understanding of COVID-19’s extended impact but also calls for a heightened awareness and proactive management of kidney health in recovering patients. As a longer follow-up period can better define long-term consequences of renal function after COVID-19, a larger prospective study will be implemented, and future research on the topic is encouraged to encompass multiple global scenarios.

### 4.2. Study Limitations

This study, aimed at assessing kidney function in Post-COVID Syndrome patients six months after SARS-CoV-2 infection, inherently faces several limitations. Firstly, its retrospective observational nature limits the ability to establish causality between COVID-19 and observed renal outcomes. The reliance on existing medical records and the potential for incomplete data raise concerns about the comprehensiveness and accuracy of the captured information. Furthermore, the exclusion criteria might have inadvertently omitted individuals with subclinical renal impairment prior to COVID-19, potentially skewing the severity of Post-COVID renal dysfunction observed. Another significant limitation is the study’s single-center design, which may limit the generalizability of the findings to broader populations, particularly considering geographical variations in COVID-19 strains and healthcare approaches. The absence of a longitudinal pre-COVID baseline for kidney function in the patients also hinders the ability to definitively attribute observed renal impairments solely to the virus. Additionally, the study’s focus on patients who have survived to discharge might introduce survivorship bias, overlooking those with severe COVID-19 who succumbed to the disease or its complications before follow-up kidney function tests could be performed. Another limitation includes the inclusion of participants with eGFR levels between 60–89 mL/min/1.73 m^2^, representing mildly decreased renal function, which may influence the purity of our cohort aimed at isolating the renal effects of Post-COVID syndrome. Lastly, the study did not account for variations in the management of acute COVID-19, such as differences in therapeutic interventions that might independently influence kidney function outcomes, nor did it fully explore the impact of COVID-19 vaccination status on renal recovery trajectories.

## 5. Conclusions

The findings underscore the significant impact of COVID-19 on long-term renal health in patients with Post-COVID Syndrome. The severity of initial infection, AKI development during admission, blood urea nitrogen increase more than 8 units above the threshold, creatinine increase with more than 23 μmol/L during admission, and urine albumin to creatinine ratio increase to more than 11 units above the threshold during COVID-19 admission were identified as key predictors of renal function decline with more than 30 units of eGFR six months post discharge. These results highlight the necessity for ongoing renal health monitoring and tailored intervention strategies to address the chronic renal implications of Post-COVID Syndrome, emphasizing the importance of considering renal outcomes in the holistic management of affected individuals.

## Figures and Tables

**Table 1:** Demographic analysis.

Variables	Control Group (*n* = 114)	Post-COVID Group (*n* = 92)	*p*-Value
Age (mean ± SD)	55.2 ± 8.5	56.9 ± 7.6	0.136
Sex			0.710
Men (n,%)	59 (51.8%)	50 (54.3%)	
Women (n,%)	55 (48.2%)	42 (45.7%)	
BMI (*n*,%)			0.900
Normal weight (18.5–24.9 kg/m^2^)	36 (31.6%)	30 (32.6%)	
Overweight (>24.9 kg/m^2^)	45 (39.5%)	38 (41.3%)	
Obese (>29.9 kg/m^2^)	33 (28.9%)	24 (26.1%)	
COVID-19 vaccination status (*n*,%)			0.317
1 dose	13 (11.4%)	12 (13.0%)	
≥2 doses	37 (32.5%)	38 (41.3%)	
Unvaccinated	64 (56.1%)	42 (45.7%)	
Antiviral medication requirement (*n*,%)			0.096
Yes	68 (59.6%)	66 (71.7%)	
No	46 (40.4%)	26 (28.3%)	
Oxygen supplementation (*n*,%)			0.620
Yes	82 (71.9%)	69 (75.0%)	
No	32 (28.1%)	23 (25.0%)	
COVID-19 severity (*n*,%)			0.909
Mild	38 (33.3%)	29 (31.5%)	
Moderate	42 (36.8%)	33 (35.9%)	
Severe	34 (29.9%)	30 (32.6%)	
Personal history (*n*,%)			
Smoking	26 (22.8%)	22 (23.9%)	0.851
CCI > 2	24 (21.1%)	20 (21.7%)	0.904
Kidney injury			
Developed AKI during admission	7 (6.1%)	16 (17.4%)	0.014
eGFR decrease <30 from baseline	11 (9.6%)	25 (27.2%)	0.001
Continuous eGFR decrease <30 at six months	–	22 (23.9%)	–

Data reported as *n* (%) and calculated using the Chi-square test and Fisher’s exact unless specified differently; BMI—Body Mass Index; SD—Standard Deviation; CCI—Charlson Comorbidity Index.

**Table 2:** Comparative analysis of blood tests between the control group and the Post-COVID group at admission.

Variables	Normal Range	Control Group during Admission (*n* = 114)	Post-COVID Group During Admission (*n* = 92)	*p*-Value
WBC (1000/mm^3^)	4.5–11.0	11.8 ± 1.7	16.6 ± 3.4	<0.001
Lymphocytes (1000/mm^3^)	1.0–4.8	2.7 ± 0.8	1.3 ± 0.6	<0.001
Hemoglobin (g/dL)	13.0–17.0	14.7 ± 1.3	13.4 ± 1.4	<0.001
AST (U/L)	10–40	21.5 ± 5.2	47.3 ± 11.8	<0.001
ALT (U/L)	7–35	18.6 ± 7.2	53.7 ± 14.3	<0.001
CRP (mg/dL)	0–10	23.1 ± 2.8	78.2 ± 24.6	<0.001
IL-6 (pg/mL)	0.8–6.4	10.3 ± 4.0	44.6 ± 19.7	<0.001
Procalcitonin (ug/L)	0–0.25	0.09 ± 0.06	0.48 ± 0.22	<0.001
D-dimers (ng/mL)	<250	285.2 ± 48.6	498.5 ± 198.4	<0.001
Ferritin (ng/mL)	20–250	292.4 ± 90.8	607.5 ± 295.7	<0.001

WBC—White Blood Cells; AST—Aspartate aminotransferase; ALT—Alanine aminotransferase; CRP—C-reactive protein; IL—Interleukin.

**Table 3:** Intragroup comparison of blood tests between the Post-COVID group at admission and six months after discharge.

Variables	Normal Range	Post-COVID Group during Admission (*n* = 92)	Post-COVID Group Six Months Post-Admission (*n* = 92)	*p*-Value
WBC (1000/mm^3^)	4.5–11.0	16.6 ± 3.4	10.9 ± 2.3	<0.001
Lymphocytes (1000/mm^3^)	1.0–4.8	1.3 ± 0.6	2.4 ± 0.7	<0.001
Hemoglobin (g/dL)	13.0–17.0	13.4 ± 1.4	14.3 ± 1.2	<0.001
AST (U/L)	10–40	47.3 ± 11.8	29.4 ± 7.5	<0.001
ALT (U/L)	7–35	53.7 ± 14.3	24.8 ± 9.1	<0.001
CRP (mg/dL)	0–10	78.2 ± 24.6	14.7 ± 9.3	<0.001
IL-6 (pg/mL)	0.8–6.4	44.6 ± 19.7	12.7 ± 4.6	<0.001
Procalcitonin (ug/L)	0–0.25	0.48 ± 0.22	0.19 ± 0.08	<0.001
D-dimers (ng/mL)	<250	498.5 ± 198.4	295.3 ± 102.7	<0.001
Ferritin (ng/mL)	20–250	607.5 ± 295.7	248.3 ± 149.2	<0.001

WBC—White Blood Cells; AST—Aspartate aminotransferase; ALT—Alanine aminotransferase; CRP—C-reactive protein; IL—Interleukin.

**Table 4:** Comparative analysis of kidney function tests between the control group and the Post-COVID group at admission.

Variables (Mean ± SD)	Normal Range	Control Group during Admission (*n* = 114)	Post-COVID Group during Admission (*n* = 92)	*p*-Value
Creatinine (μmol/L)	60–110	84.5 ± 11.7	109.7 ± 16.4	<0.001
eGFR (mL/min/1.73 m^2^)	>90	91.2 ± 7.3	65.3 ± 12.8	<0.001
BUN (mg/dL)	7–20	15.2 ± 3.6	23.7 ± 7.2	<0.001
Proteinuria (mg/dL)	<150	105.1 ± 48.6	198.3 ± 91.2	<0.001
Hematuria (cells/HPF)	0–3	1.5 ± 1.2	3.8 ± 2.1	<0.001
Albuminuria (mg/g)	<30	22.7 ± 8.2	52.4 ± 18.9	<0.001
ACR (mg/g)	<30	24.8 ± 10.1	59.1 ± 23.6	<0.001
Urine specific gravity	1.005–1.030	1.021 ± 0.004	1.016 ± 0.008	<0.001
Urine osmolality (mOsm/kg)	300–900	600.2 ± 140.3	535.7 ± 195.6	0.006
Sodium (mmol/L)	135–145	139.8 ± 3.9	138.2 ± 4.5	0.138
Potassium (mmol/L)	3.5–5.1	4.3 ± 0.5	4.7 ± 0.6	<0.001

SD—Standard Deviation; ACR—Urine Albumin to Creatinine Ratio; BUN—Blood Urea Nitrogen; eGFR—Estimated Glomerular Filtration Rate.

**Table 5:** Intragroup comparison of kidney function tests between the Post-COVID group at admission and six months after discharge.

Variables (Mean ± SD)	Normal Range	Post-COVID Group during Admission (*n* = 92)	Post-COVID Group Six Months Post-Admission (*n* = 92)	*p*-Value
Creatinine (μmol/L)	60–110	109.7 ± 16.4	90.4 ± 13.6	<0.001
eGFR (mL/min/1.73 m^2^)	>90	65.3 ± 12.8	70.6 ± 11.1	0.002
BUN (mg/dL)	7–20	23.7 ± 7.2	18.3 ± 5.8	<0.001
Proteinuria (mg/dL)	<150	198.3 ± 91.2	155.9 ± 65.4	0.002
Hematuria (cells/HPF)	0-3	3.8 ± 2.1	2.0 ± 1.7	<0.001
Albuminuria (mg/g)	<30	52.4 ± 18.9	38.8 ± 15.2	<0.001
ACR (mg/g)	<30	59.1 ± 23.6	39.5 ± 16.3	<0.001
Urine specific gravity	1.005–1.030	1.016 ± 0.008	1.022 ± 0.005	<0.001
Urine osmolality (mOsm/kg)	300–900	485.7 ± 195.6	645.8 ± 170.2	<0.001
Sodium (mmol/L)	135–145	138.2 ± 4.5	140.1 ± 3.6	<0.001
Potassium (mmol/L)	3.5–5.1	4.7 ± 0.6	4.5 ± 0.4	0.007

SD—Standard Deviation; ACR—Urine Albumin to Creatinine Ratio; BUN—Blood Urea Nitrogen; eGFR—Estimated Glomerular Filtration Rate.

**Table 6:** Comparison of kidney function tests between the control group at admission and the Post-COVID group at six months post admission.

Variables (Mean ± SD)	Normal Range	Control Group during Admission (*n* = 114)	Post-COVID Group Six Months Post-Admission (*n* = 92)	*p*-Value
Creatinine (μmol/L)	60–110	84.5 ± 11.7	90.4 ± 13.6	0.001
eGFR (mL/min/1.73 m^2^)	>90	91.2 ± 7.3	70.6 ± 11.1	<0.001
BUN (mg/dL)	7–20	15.2 ± 3.6	18.3 ± 5.8	<0.001
Proteinuria (mg/dL)	<150	105.1 ± 48.6	155.9 ± 65.4	<0.001
Hematuria (cells/HPF)	0–3	1.5 ± 1.2	2.0 ± 1.7	0.014
Albuminuria (mg/g)	<30	22.7 ± 8.2	38.8 ± 15.2	<0.001
ACR (mg/g)	<30	24.8 ± 10.1	39.5 ± 16.3	<0.001
Urine specific gravity	1.005–1.030	1.021 ± 0.004	1.022 ± 0.005	0.180
Urine osmolality (mOsm/kg)	300–900	600.2 ± 140.3	645.8 ± 170.2	0.036
Sodium (mmol/L)	135–145	139.8 ± 3.9	140.1 ± 3.6	0.289
Potassium (mmol/L)	3.5–5.1	4.3 ± 0.5	4.5 ± 0.4	0.005

SD—Standard Deviation; ACR—Urine Albumin to Creatinine Ratio; BUN—Blood Urea Nitrogen; eGFR—Estimated Glomerular Filtration Rate.

**Table 7:** Regression analysis of factors predicting continuous eGFR decrease <30 at six months post SARS-CoV-2 infection.

Independent Variables	Coefficient (β)	Std. Error	*p*-Value	95% CI
Age	−0.24	0.08	0.004	(−0.40, −0.08)
COVID-19 Severity (Severe = 1)	2.05	0.59	0.001	(1.89, 4.21)
Developed AKI (Yes = 1, No = 0)	3.47	0.76	<0.001	(1.97, 4.97)
Creatinine increase (>23 μmol/L above NR)	2.21	0.65	<0.001	(1.93, 3.49)
ACR increase (>11 mg/g above NR)	1.88	0.47	<0.001	(1.06, 4.80)
BUN increase (>8 mg/dL above NR)	1.34	0.38	0.001	(1.10, 5.08)

CI—Confidence Interval; BUN—Blood Urea Nitrogen; NR—Normal Range; ACR—Urine Albumin to Creatinine Ratio.

## Data Availability

Data available on request from the author.
